# Pancreatoscopy-Guided Lithotripsy for Pancreatic Duct Stones: A Systematic Review and Meta-Analysis

**DOI:** 10.5152/tjg.2024.24110

**Published:** 2024-11-01

**Authors:** Peiyao Huang, Hayat Khizar, Wensong Song, Jianfeng Yang

**Affiliations:** 1 Department of Gastroenterology, The Fourth Affiliated Hospital, International Institutes of Medicine, Zhejiang University School of Medicine, Yiwu, Zhejiang, China; 2 Department of Surgery, The Fourth Affiliated Hospital, International Institutes of Medicine, Zhejiang University School of Medicine, Yiwu, Zhejiang, China; 3 Department of Gastroenterology, Hangzhou First People’s Hospital, Zhejiang University School of Medicine, Hangzhou, Zhejiang, China; 4 Key Laboratory of Integrated Traditional Chinese and Western Medicine for Biliary and Pancreatic Diseases of Zhejiang Province, Hangzhou, Zhejiang, China; 5 Key Laboratory of Clinical Cancer Pharmacology and Toxicology Research of Zhejiang Province, Hangzhou, Zhejiang, China

**Keywords:** Electrohydraulic lithotripsy, laser lithotripsy, pancreatoscopy, pancreatic duct stones, meta-analysis, review

## Abstract

**Background/Aims:**

Pancreatic duct stones (PDS) are one of the leading complications of chronic pancreatitis, causing intractable upper abdominal pain, aggravating the underlying disease, and even increasing the risk of pancreatic cancer. At present, pancreatoscopy-guided lithotripsy is considered the second-line endoscopic treatment for pancreatic duct stones. In this systematic review and meta-analysis, we evaluated the efficacy and safety of pancreatoscopy-guided lithotripsy.

**Materials and Methods:**

A systematic search was conducted across several medical electronic databases, including PubMed, Web of Science, Medline, and Embase, encompassing publications up to December 2022. Studies reporting complete stone clearance rate, clinical success rate, and adverse event rate were included for analysis. We further aimed to compare the outcomes between electrohydraulic lithotripsy and laser lithotripsy treatment groups.

**Results:**

A total of 17 studies (5 prospective studies and 12 retrospective studies) with 441 patients were included in the meta-analysis. Pooled complete stone clearance rate was 81% (95% CI, 0.74-0.88), pooled clinical success rate was 90% (95% CI, 0.84-0.95), while the pooled adverse event rate was 12% (95% CI, 0.07-0.19).

**Conclusion:**

Pancreatoscopy-guided lithotripsy is a safe and effective treatment for pancreatic duct stones. This is evidenced by high pooled rates of complete stone clearance and clinical success, combined with a relatively low incidence of adverse events.

**Main Points**

- Pancreatoscopy-guided lithotripsy is a safe and effective treatment for pancreatic duct stones.
- Pancreatoscopy-guided lithotripsy demonstrated a high complete stone clearance rate, reaching 81% in this meta-analysis.
- In this meta-analysis, pancreatoscopy-guided lithotripsy achieved a high clinical success rate of 90%.
- In this meta-analysis, pancreatoscopy-guided lithotripsy exhibited a favorable safety profile, with a relatively low adverse event rate of 12%.

## Introduction

Chronic Pancreatitis (CP) is a progressive chronic inflammatory disease of pancreatic tissue caused by a combination of genetic, environmental, and other causes. Pancreatic Duct Stones (PDS) are one of the major complications of CP. PDS have a predominantly inorganic composition, with calcium carbonate as the primary constituent.^1^ Organic components are also present and include pancreatic stone protein (PSP), trypsinogen, lactoferrin, amylase, and fragments of pancreatic exocrine cells.^2,3^ Chronic obstruction by pancreatic duct stones can lead to impaired drainage of pancreatic fluid, resulting in elevated pressure within the pancreatic duct and interstitium. Subsequently, this may also contribute to the development of severe abdominal pain. Furthermore, it can induce repeated attacks of pancreatitis, and cause ischemia, fibrosis, and local perineural inflammation of pancreatic tissue. Over time, it will not only aggravate the condition of CP but also may increase the risk of pancreatic cancer.^4,5^ One of the most important treatments for PDS is removing stones in the pancreatic duct to reduce pressure and alleviate clinical symptoms. Currently, treatment of PDS includes medical therapy, endoscopic interventions, and surgical resection. Pancreatoscopy-guided lithotripsy is another recommended endoscopic treatment when ESWL is not available or for stones that were not fragmented after adequately performed ESWL.^6^ With the development of this technology, pancreatoscopy-guided lithotripsy and its application in the management of pancreatic duct stones has become increasingly widespread. We performed a systematic review and meta-analysis to evaluate the efficacy and safety of pancreatoscopy-guided lithotripsy for pancreatic duct stones.

## Materials and Methods

We followed the PRISMA (Preferred Reporting Items for Systematic Reviews and Meta-Analyses) guidelines for reporting this systematic review and meta-analysis.^7^ The literature retrieval, data extraction, and quality assessment processes were independently conducted by two authors. Disagreements were resolved through consultation with the third author.

### Search Strategy

A comprehensive search strategy was employed across multiple electronic databases, including PubMed, Web of Science, Medline, and Embase. Publications up to December 2022 were included in the study. Keywords “pancreatic calculi,” “pancreatic duct stones,” “pancreatolithiasis,” “PDS,” “pancreatoscopy,” and “Spyglass” were used for Boolean logic operations.

### Study Selection

**Inclusion Criteria**: Studies that used pancreatoscopy-guided lithotripsy in patients with PDS.

**Exclusion Criteria:** (1) Studies in which data or full text cannot be obtained; (2) Review articles, case reports, conference abstracts, letters, book chapters, comments, animal studies, and other non-clinical research literature; (3) Repeated publications of literature.

### Data Extraction

The following relevant data were obtained from the literature: year of publication, country, type of study, study duration, number of patients, gender, age, etiology of CP, number of stones, size of stones, location of stones, pancreatoscopy model, type of lithotripsy, operation duration, intervention frequency, complete stone clearance rate, clinical success rate, incidence of adverse events, and specific adverse events.

### Outcome and Definitions

The primary outcome of this study was the complete stone clearance rate. Complete stone clearance was defined as achieving 100% removal of PDS through pancreatoscopy-guided lithotripsy, which includes electrohydraulic lithotripsy (EHL), laser lithotripsy (LL), or other lithotripsy devices. Secondary outcomes included the clinical success rate and adverse event rate. Clinical success was defined as the resolution or significant improvement in symptoms during follow-up, as evidenced by a reduction of at least 50% in opioid use, pain score, or hospital length of stay. Adverse events (AE) were defined as any events that affected the patient’s clinical course and/or resulted in readmission to the hospital or prolongation of existing hospitalization. These events mainly included postoperative pancreatitis, abdominal pain, fever, bleeding, contrast extravasation, and perforation.^8^

### Quality Assessment

The literature quality of the included studies was evaluated using the Methodological Index for Non-Randomized Experimental Studies (MINORS).^9^ There were 12 evaluation indicators in total, and each indicator was marked with 0~2 points. 0 points means no report, 1 point means reported but insufficient, and 2 points means reported and sufficient. The quality of each study was categorized according to the total score of the study: poor (0-5 points), average (6-10 points), and good (11-16 points).

### Statistical Analysis

Stata software version 17.0 (College Station, TX: StataCorp LLC) was used for statistical analysis and mapping of related results. Categorical variables were displayed as counts and percentages. Data adhering to a normal distribution were described using mean ± SD, while data not normally distributed were depicted through median (IQR). First, an analysis of the technical success rate, clinical success rate, and adverse event occurrence rate of all studies was conducted. This was followed by a subgroup analysis according to the type of laser used. In the test of heterogeneity, if *I* ^*2*^ ≤ 50%, there was no significant heterogeneity, so a fixed-effect model combined effect size analysis was used. If *I* ^*2*^ > 50%, the heterogeneity of the included studies was large, and the random effects model was used for analysis. Statistical significance was set at *P* < .05.

## Results

### Search Results, Study Characteristics, and Evaluation

**Figure 1.**
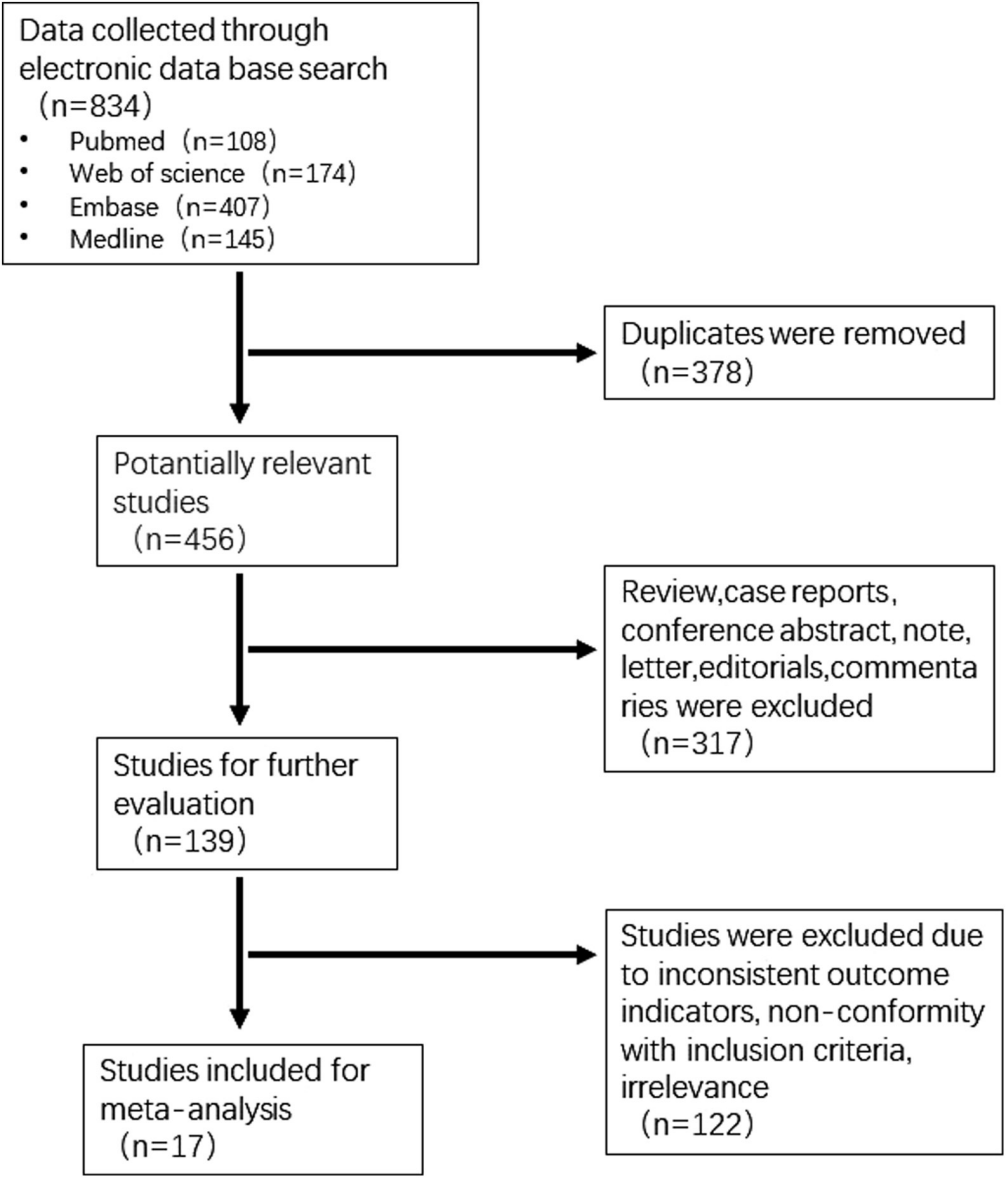
Flowchart of included studies.

**Supplementary Figure 1.**
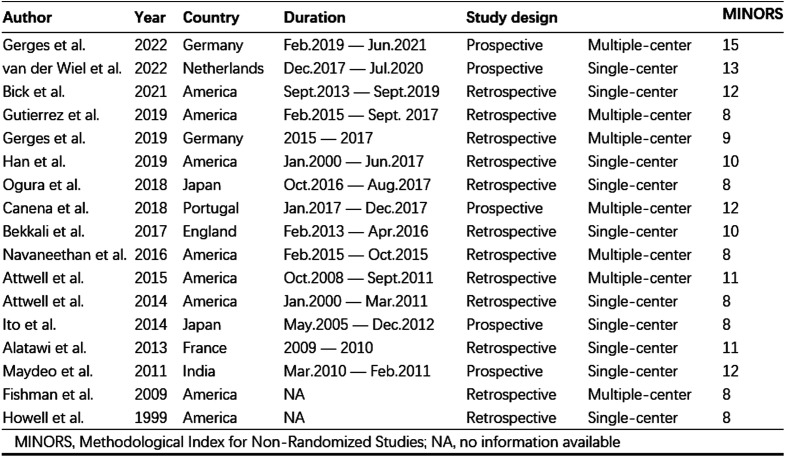
Basic Characteristics of Study.

A total of 834 relevant studies were retrieved, including 108 from PubMed, 174 from Web of Science, 145 from Medline, and 407 from Embase. Then, 378 duplicate studies and 317 non-clinical research studies were excluded. After reading the abstract or full text, 122 studies, including inconsistent outcome indicators, non-inclusion criteria, and irrelevant to this research theme, were excluded. Following the selection process, 17 studies were deemed eligible for inclusion in the systematic review (Figure 1). The included studies consisted of 5 prospective studies and 12 retrospective studies. There were 10 single-center studies and 7 multi-center studies. All the studies lasted more than 1 year (Supplementary Figure 1). A total of 441 patients were included from the 17 studies. Analysis of baseline patient demographics revealed a predominance of alcohol-induced chronic pancreatitis. Additionally, the head of the pancreas emerged as the most common site for stone formation (Table 1). A quality assessment of the 17 included studies yielded a mean score of 10.11. Notably, 7 studies were categorized as good-quality, while the remaining 10 studies were classified as medium-quality. Based on the above analysis, the quality of included studies was acceptable (Supplementary Figure 1).

### Primary Outcomes

#### Complete Clearance of Stones:

**Figure 2.**
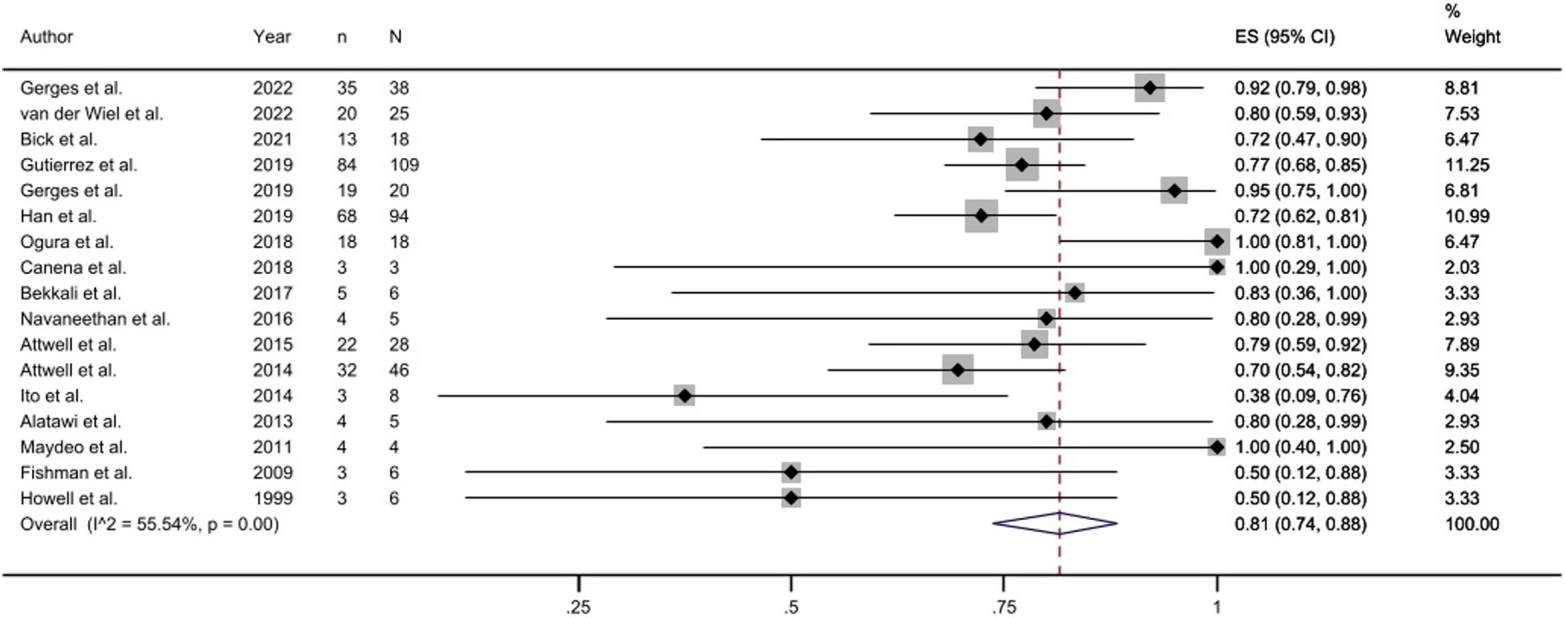
Forest plot of complete clearance of stones.

**Supplementary Figure 2.**
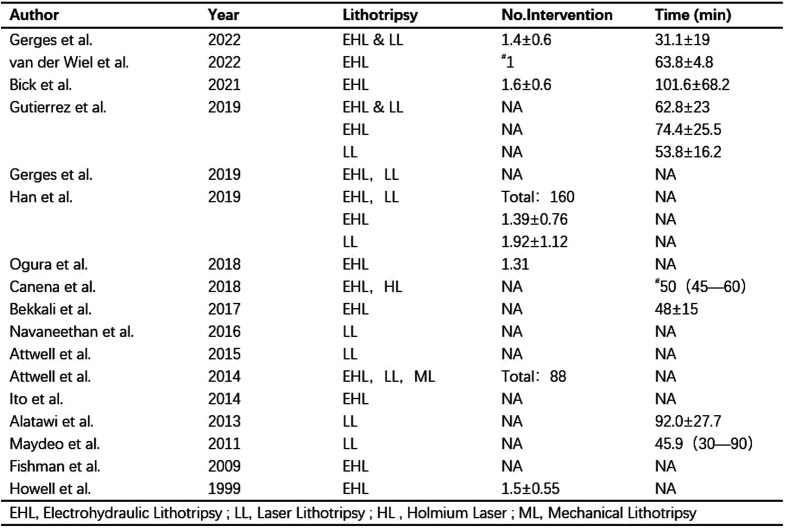
Basic information about the operation.

Studies have reported complete stone clearance rates ranging from 38% to 100%. Meta-analysis of the random effects model showed that the complete stone clearance rate of pancreatoscopy-guided lithotripsy was 81% (95% CI, 0.74-0.88, Figure 2). Eight studies reported the specific operation time (Supplementary Figure 2). According to the formula, the interval between median and quartile was converted to mean and standard deviation.^10^ After analysis, the average operation time was 60.45 ± 33.39 minutes.

### Secondary Outcomes

#### Clinical Success:

**Figure 3.**
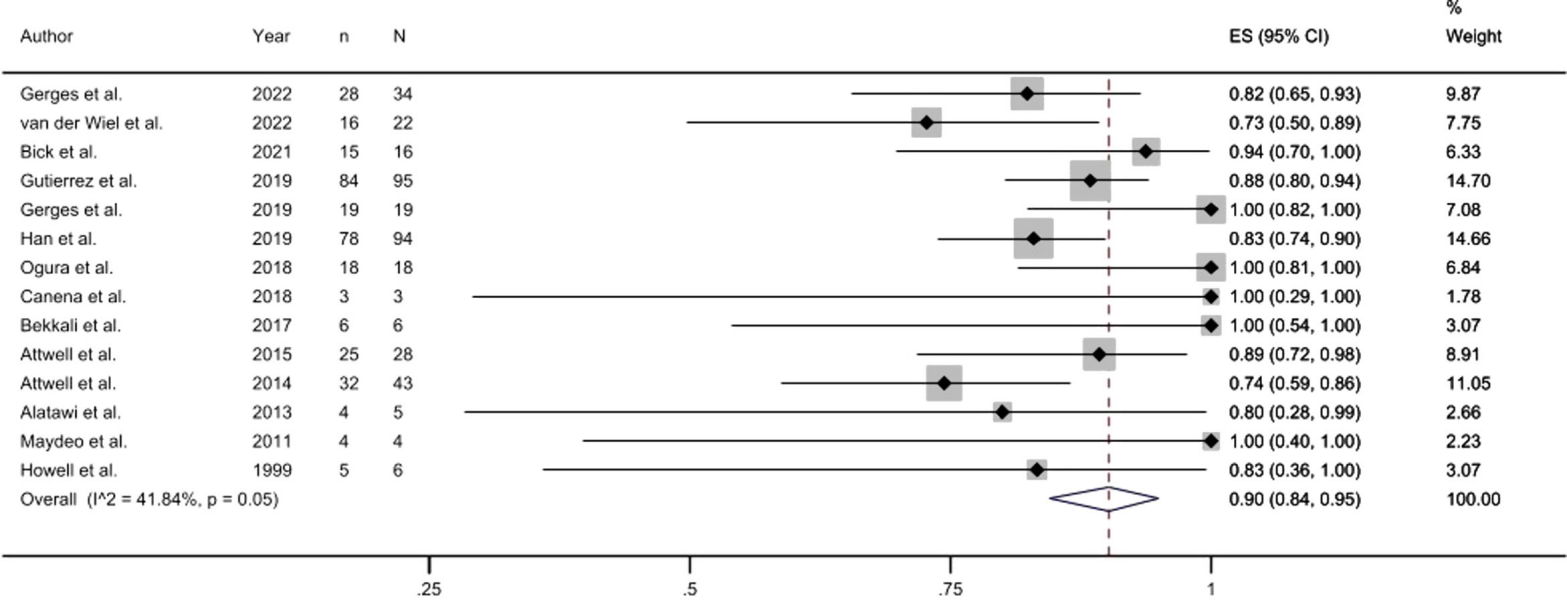
Forest plot of clinical success.

Fourteen of the included studies provided data on the clinical success rate of pancreatoscopy-guided lithotripsy. Pooled analysis of these studies revealed a success rate of 90% (95% CI, 0.84-0.95). Notably, 5 studies reported success rates of up to 100% (Figure 3).

### Adverse Events

**Figure 4.**
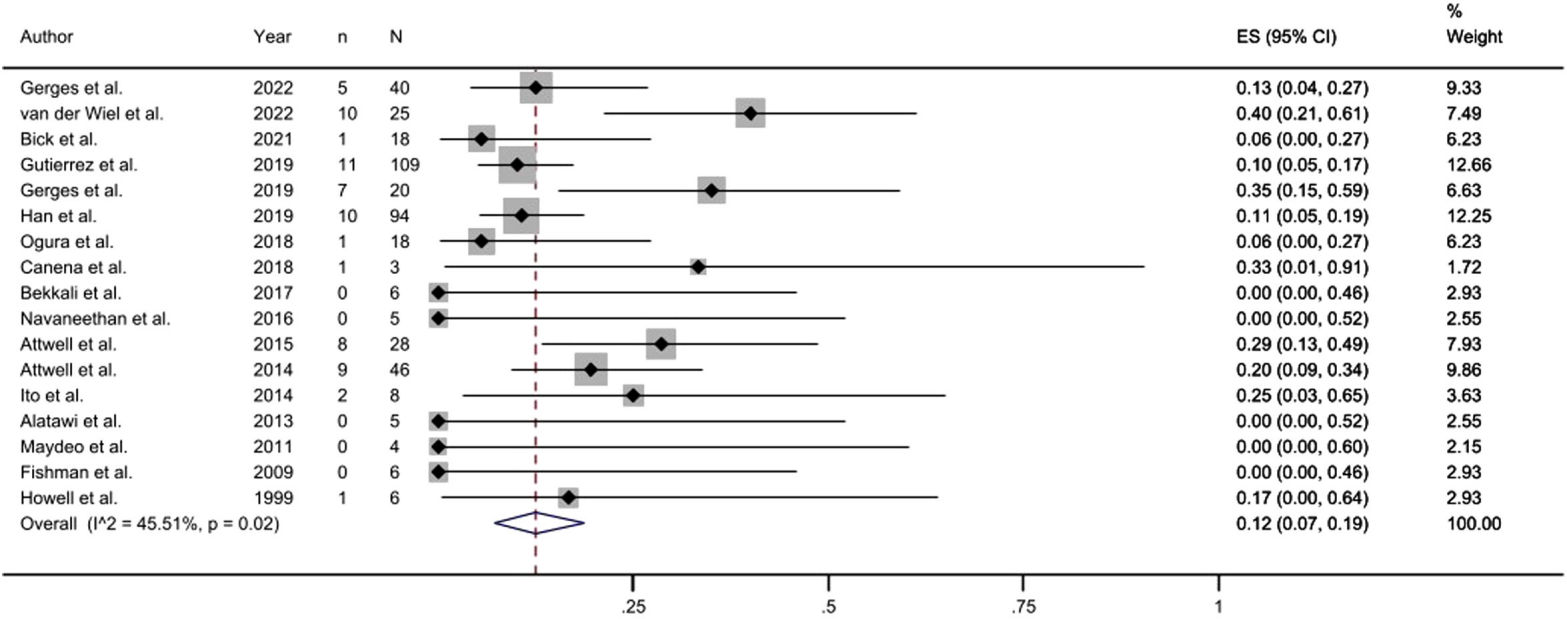
Forest plot of adverse events.

Among the 17 studies included, the pooled AE rate was 12% (95% CI, 0.07-0.19, Figure 4). In 5 small sample studies (number of patients <15), the incidence of AE was 0. According to the statistics of specific AE, post-endoscopic retrograde cholangiopancreatography pancreatitis (PEP) was the most frequent complication, accounting for 47% of all AEs, while the pooled incidence was 8% after meta-analysis. Abdominal pain was the second most frequent complication, accounting for 35% of all AEs. Other complications, such as perforation, bleeding, and fever, occurred in a small number of patients (Table 2). Literature reports suggest that these complications are typically mild in most patients.

### Comparison of EHL and LL

**Figure 5.**
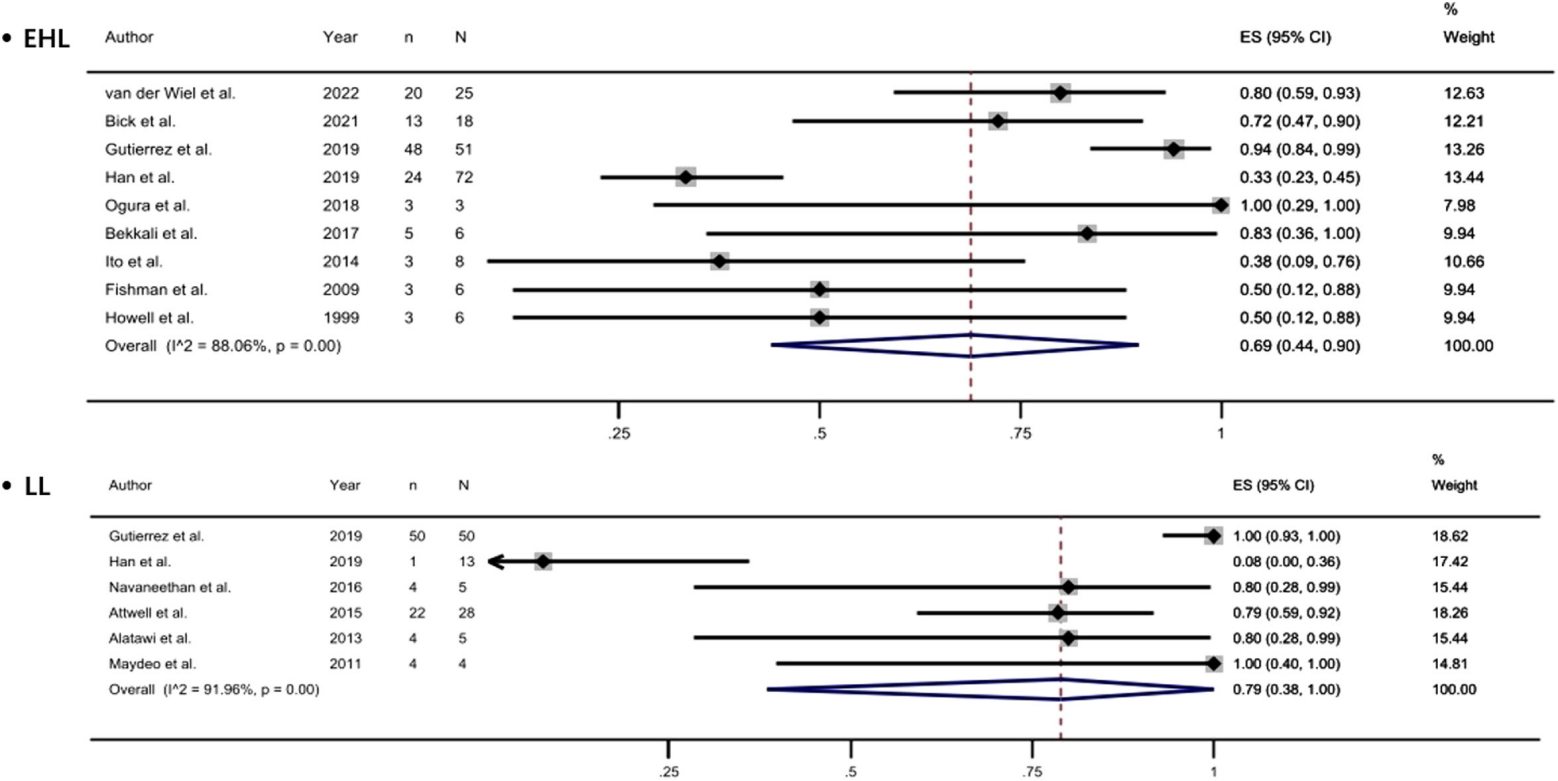
Forest plot of complete stone clearance with using EHL and with using LL.

**Supplementary Figure 3.**
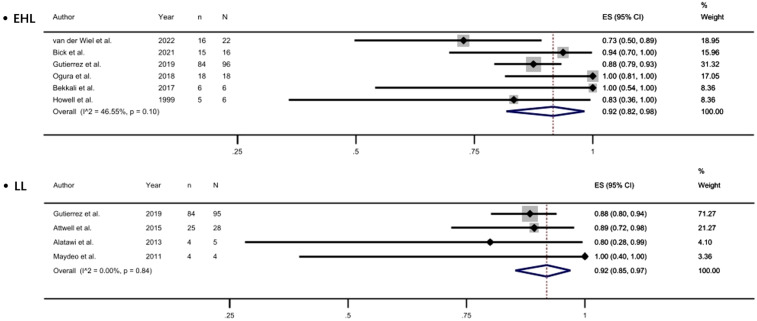
Forest plot of clinical success with using EHL and with using LL.

**Supplementary Figure 4.**
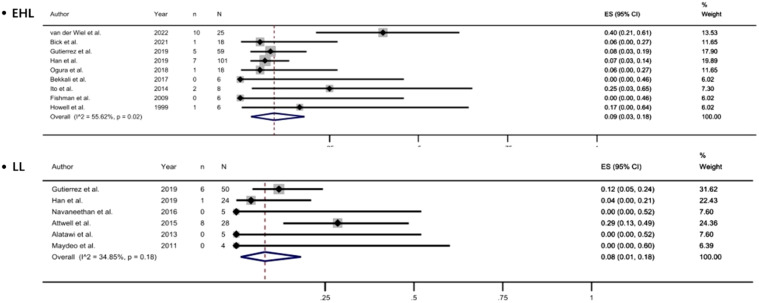
Forest plot of adverse events with using EHL and with using LL.

Currently, the two most commonly used lithotripsy techniques are EHL and LL. We analyzed the efficacy and safety of EHL and LL in the treatment of PDS. With EHL, the complete clearance rate of PDS was 69%, the clinical success rate was 92%, while the incidence of AE was 9%. With LL, the complete clearance rate was 79%, the clinical success rate was 92%, and an AE rate was 8% (Figure 5, and Supplementary Figures 3-4).

## Discussion

This meta-analysis investigated the efficacy and safety of pancreatoscopy-guided lithotripsy for the management of PDS by evaluating data from 17 studies. Our analysis revealed that the pooled complete stone clearance rate was 81%, with the pooled clinical success rate of 90% and pooled adverse event rate of 12%. When employing EHL, the complete stone clearance rate was 69%, the clinical success rate was 92%, and the AE rate was 9%. With the use of LL, these rates were 79% for complete clearance, 92% for clinical success, and 8% for AE.

Endoscopic Retrograde Cholangiopancreatography (ERCP) plays an important role in hepatobiliary pancreatic diseases and is currently an effective treatment for bile duct stones. However, compared with bile duct stones, PDS are harder and often embedded in the pancreatic duct, significantly increasing the difficulty of stone extraction. Due to the inherently small caliber of the pancreatic duct (3-4 mm), compared to the bile duct, ERCP poses a significant challenge in patients with chronic pancreatitis. These patients often have concomitant pancreatic duct strictures or anatomic variations that further impede guidewire cannulation during traditional ERCP, resulting in suboptimal outcomes for stent placement. A recent retrospective analysis revealed a low stone clearance rate of 25.71% following ERCP alone. Additionally, the one-year pain relief rate was modest at 62.86%.^11^ The study by Sauerbruch et al in 1987 described the first application of extracorporeal shock wave lithotripsy (ESWL) in conjunction with ERCP for the management of patients with PDS.^12^ This combined approach has gained increasing usage among gastroenterologists due to its potential advantages. ESWL utilizes shock waves to fragment pancreatic duct stones. Three types of ESWL exist based on the shock wave source: hydraulic, piezoelectric, and electromagnetic.^13^ By successfully crushing stones into smaller fragments, ESWL facilitates their subsequent self-discharge or removal by physicians, ultimately improving treatment success rates.

A meta-analysis by van Huijgevoort et al demonstrated an 86.3% stone fragmentation rate following ESWL in conjunction with ERCP. This approach achieved a complete stone clearance rate of 69.8%.^14^ The latest ESGE guidelines recommend ESWL for the fragmentation of radiopaque, obstructive main pancreatic duct (MPD) stones exceeding 5 mm in diameter, located within the pancreatic head or body. However, the widespread adoption of ESWL is limited by the availability of lithotripsy devices at many healthcare facilities due to its high cost. Furthermore, inherent energy dissipation during shock wave propagation through intervening tissues can lessen its efficacy.^6^

In recent years, technologies of pancreatoscopy have continued to develop, providing a new and effective protocol for PDS. In 1976, Kawai et al introduced a novel technique termed peroral choledocho-pancreatoscopy.^15^ This approach utilizes a thin, flexible fiberscope (often referred to as a “baby scope”) that can be inserted through the working channel of a modified duodenoscope (the “mother scope”) to directly visualize the common bile duct and pancreatic duct.^16,17^ Following the introduction of peroral choledocho-pancreatoscopy, the SpyGlass peroral cholangioscopy (SpyGlass Direct Visualization System, Boston Scientific, MA) emerged in 2007 as a minimally invasive tool for clinical use. This system incorporates a design featuring four-way deflection steering and a dedicated irrigation channel. These features facilitate direct visualization of the biliary and pancreatic ductal anatomy, enabling tissue acquisition for biopsy and facilitating stone fragmentation procedures.^18^ In 2015, Boston Scientific introduced a digital version of the system, the SpyGlass DS, offering significant advancements in image quality.^19^

Two most commonly used lithotripsy techniques are EHL and LL. Electrohydraulic lithotripsy (EHL) employs a pair of coaxially insulated electrodes that generate high-voltage electrical discharges to create a powerful hydraulic shockwave, effectively fragmenting calculi. In contrast, LL utilizes a laser beam to deliver precisely targeted pulses of laser energy. These laser pulses produce a mechanical shockwave resulting in fragmentation. A 1.9-French to 3-French EHL or LL probe can be introduced through the working channel of the pancreatoscope, typically measuring 1.2 mm in diameter. These probes facilitate the fragmentation of pancreatic duct stones within the working channel.^20,21^ While our meta-analysis revealed similar clinical success rates for both EHL and LL, we observed potential advantages associated with LL in terms of efficacy and safety. Several comparative studies employing indirect analyses have suggested potentially superior clinical outcomes associated with LL compared to other techniques.^20,22^ Gutierrez et al investigated the efficacy of EHL and LL in a multicenter, retrospective study. Their analysis of patients treated with each modality under pancreatoscopy revealed a shorter treatment duration and a trend towards a higher technical success rate with LL compared to EHL. However, this difference did not reach statistical significance. Interestingly, EHL appeared to be associated with a lower incidence of adverse events.^23^ A comparative study by Han et al demonstrated a significantly higher complete stone clearance rate following EHL compared to LL. However, EHL was also associated with a higher incidence of adverse events, although this difference was not statistically significant. It is noteworthy that the study has a significant imbalance in sample size, with a considerably larger patient population assigned to the EHL group.^24^ The study by Guzmán-Calderón et al reported a higher success rate with EHL compared to LL. However, this difference did not reach statistical significance, leaving the comparative efficacy and safety of these modalities inconclusive.^25^ To definitively address this uncertainty, future research efforts should prioritize well-designed, large-scale randomized controlled trials.

Previous studies have suggested that alcohol is the most common cause of CP in developed countries. Our meta-analysis included studies conducted within developed countries, encompassing the United States, Japan, Germany, France, the Netherlands, and Portugal. Among the etiologies of pancreatitis investigated, alcoholic pancreatitis emerged as the most prevalent cause. In contrast, data from developing countries remains scarce, limiting our understanding of the disease burden in these regions. In this meta-analysis, the complete stone clearance rate of PDS treated with pancreatoscopy-guided lithotripsy was 81%. This result benefits from the rigorous quality assessment of prior studies and the strict selection criteria employed for the complete stone clearance rate outcome. However, the inclusion of eight studies with limited sample sizes (fewer than 15 patients) introduces a potential bias due to the increased influence of errors within these studies. Furthermore, previous studies have demonstrated the utility of pre-operative pancreatic duct stent placement for catheter decompression. However, the additional benefit of such stenting on stone clearance remains uncertain and requires further investigation.^26,27^ Several factors beyond stone composition influence the stone clearance rate during pancreatoscopy. These include the operator’s experience, stone size and location within the pancreatic ductal system, the severity of stenosis of the pancreatic duct, and the angulation of the MPD. Notably, the study by Gutierrez et al demonstrated that the presence of more than three stones was the only factor consistently associated with the need for repeat procedures.^23^

Pancreatic duct stones (PDS) typically manifest with recurrent episodes of upper abdominal pain as the most prominent symptom. Other clinical features may include fatty diarrhea, abdominal distension, obstructive jaundice, and weight loss. Within the studies we reviewed, assessment of clinical success typically depends on the resolution of abdominal pain and a reduction in hospital length of stay. Our analysis revealed a significantly higher clinical success rate (90%) compared to the complete stone clearance rate. This observation suggests that even partial stone removal might effectively alleviate the elevated pressure within the pancreatic duct and interstitium, leading to variable degrees of abdominal pain improvement in most patients. It is important to acknowledge, however, that limitations exist in the assessment of clinical success rates. The studies included in our analysis showed inconsistencies in the application of pain scoring criteria. Additionally, inherent variations in patient pain tolerance contribute to subjectivity in the evaluation process. Furthermore, the absence of standardized discharge criteria raises concerns about the potential overestimation of clinical success rates. Despite stone removal, a small subset of patients continues to experience abdominal pain. This persistence of pain may be attributed to the incomplete understanding of PDS pathogenesis. While factors like inflammation, biliary diseases, hypercalcemia, autoimmune conditions, genetic mutations, and even age can influence the course of PDS, stone removal itself might not demonstrably improve these underlying contributors.^28-30^ Furthermore, chronic inflammation associated with long-standing CP may contribute to peripheral and central nervous system sensitization. This can manifest as visceral hypersensitivity, allodynia and hyperalgesia.^31^ Notably, endoscopic interventions for such pain mechanisms have demonstrated encouraging results in the treatment of pancreaticobiliary diseases.^32,33^

Pancreatoscopy-guided lithotripsy carries a risk of certain AE, including acute pancreatitis, abdominal pain, fever, post-sphincterotomy bleeding, contrast extravasation, and perforation. Our analysis revealed a 12% incidence of AE following pancreatoscopy, suggesting a relatively safe procedure with some limitations. The most frequent complication observed was PEP, with a pooled incidence rate of 8% identified through the meta-analysis. According to the ASGE guideline, PEP occurs in approximately 8% of moderate-risk procedures and 15% of high-risk procedures.^34^ Our findings support the data that pancreatoscopy itself does not substantially elevate the risk of PEP. This observation aligns with recommendations from other studies, which encourage the use of guidewire intubation, pancreatic duct stents, aggressive intravenous fluid resuscitation, and rectal indomethacin administration to minimize the incidence of PEP.^34-37^ While our analysis did not identify recommendations for these measures within the included studies, their potential effectiveness in reducing PEP warrants further investigation. Furthermore, recent advancements in pancreatoscopy may allow improved visualization during the procedure, potentially minimizing the risk of puncture, bleeding, and ductal injury. It is important to note that the reported adverse events were generally mild in severity, prompting physicians to prioritize symptomatic management.

Our study is subject to several limitations. First, heterogeneity was observed in the reporting of the included studies, with some lacking essential data such as age, sex, and adverse event profiles. Secondly, an important limitation is the lack of comparative studies directly evaluating EHL and LL against each other. Despite these limitations, our analysis yielded sufficient data to support the efficacy of both pancreatoscopy-guided EHL and LL in the treatment of PDS.

Pancreatoscopy-guided lithotripsy is a promising therapeutic modality for managing PDS, demonstrating high success rates with relatively low AEs. This minimally invasive approach has the potential to be the first-line treatment for PDS in the field of endoscopy. Future research efforts should prioritize optimizing equipment selection and procedural techniques to further minimize complication rates. Additionally, well-designed comparative studies are needed to definitively establish the comparative efficacy of pancreatoscopy-guided lithotripsy to alternative treatments such as ESWL.

## Figures and Tables

**Table 1.** Basic Information About the Patients

Study	Year	No. of Patients (Male/Female)	Age	Etiology	No. of Stones	Stone Size (mm)	Stone Location
Gerges et al^38^	2022	40 (22/18)	56.7 ± 15.5	Idiopathic 23; alcohol 14; metabolic 1; hereditary 1; abnormal anatomy 1	1.7 ± 1.3	9.8 ± 3.5	Head 24; body 14; corpus 5
van der Wiel et al^27^	2022	34 (21/13)	56.7 ± 13.5	Idiopathic 10; alcohol 20; abnormal anatomy 2; hypercalcemia 2	1 (1-3)	8.6 ± 3.3	Head 23; neck 2
Bick et al^39^	2021	18 (9/9)	61.3 ± 11.7	*Idiopathic 4; alcohol 10; smoke 13; hereditary 2; obstructive 1	1.7 ± 1.0	10.3 ± 7.4	Head 17; body 6; neck 2; caudal 1
Gutierrez et al^23^	2019	109 (77/32)	54.7 ± 15.0	NA	NA	< 10 mm, 62 10-19 mm, 32 > 20 mm, 15	Head 54; neck 23; body 15; caudal 6; multiposition 11
Gerges et al^40^	2019	20 (11/9)	62.4 ± 14.8	NA	1.9 ± 1.2	9.3 ± 2.5	Head 8; corpus 10; caudal 4
Han et al^24^	2019	94 (NA)	NA	NA	1个, 19; 2-4个, 49; ≥ 5个, 26	8.9 ± 5.3	Head 84; body 6; caudal 4
Canena et al^41^	2019	3 (3/0)	NA	NA	2 (1-3)	6 (5-7)	Head 1; body 2
Ogura et al^42^	2018	18 (15/6)	NA	Idiopathic 4; alcohol 10	NA	12^#^	Head 8; body 14; caudal 2
Bekkali et al^43^	2017	6 (3/3)	45 ± 7	NA	NA	10.6 ± 3.9	Head 6; caudal 1
Navaneethan et al^44^	2016	5 (NA)	NA	NA	NA	9	NA
Attwell et al^45^	2015	28 (16/12)	51^#^	Idiopathic 9; alcohol 14; other 5	NA	NA	Head 9; neck 3; body 9; caudal 1; multiposition 6
Attwell et al^46^	2014	46 (23/23)	58^#^	Idiopathic 11; alcohol 26; other 9	NA	8^#^	Head 32; body 32; caudal 4
Ito et al^47^	2014	8 (NA)	NA	NA	NA		Head 8
Alatawi et al^48^	2013	5 (4/1)	53 ± 9.1	Alcohol 4; abnormal anatomy 1	NA	7.6 ± 2.5	Head 4; neck 1
Maydeo et al^49^	2011	4 (3/1)	NA	NA	NA	5.6 (5.0-6.0)	Head 2; corpus 1; multiposition 1
Fishman et al^50^	2009	6 (NA)	NA	NA	NA	5-14	Head 6
Howell et al^51^	1999	6 (5/1)	61.17 ± 12.37	NA	NA	NA	NA

NA, no information available.

^#^Median.

*Patients may have a concurrent etiology.

**Table 2.** Adverse Events in all Studies

Study	Year	Total	PEP	Bleeding	Perforation	Abdominal Pain	Cholangitis	Fever
Gerges et al^38^	2022	5	1	0	0	2	0	0
van der Wiel et al^27^	2022	10	7	0	0	2	1	0
Bick et al^39^	2021	1	1	0	0	0	0	0
Gutierrez et al^23^	2019	11	5	2	1	3	0	3
Gerges et al^40^	2019	7	6	1	0	0	0	0
Han et al^24^	2019	10	2	0	0	5	0	0
Canena et al^41^	2019	1	0	0	0	1	0	0
Ogura et al^42^	2018	1	1	0	0	0	0	0
Bekkali et al^43^	2017	0	0	0	0	0	0	0
Navaneethan et al^44^	2016	0	0	0	0	0	0	0
Attwell et al^45^	2015	8	1	0	0	7	0	0
Attwell et al^46^	2014	9	6	0	1	2	0	0
Ito et al^47^	2014	2	1	0	0	0	0	0
Alatawi et al^48^	2013	0	0	0	0	0	0	0
Maydeo et al^49^	2011	0	0	0	0	0	0	0
Fishman et al^50^	2009	0	0	0	0	0	0	0
Howell et al^51^	1999	1	0	0	0	1	0	0

PEP, post-endoscopic retrograde cholangiopancreatography pancreatitis.

## Data Availability

The data that support the findings of this study are available on request from the corresponding author.
