# Supplementary material for: Pancreatoscopy-Guided Lithotripsy for Pancreatic Duct Stones: A Systematic Review and Meta-Analysis
Source: Turk J Gastroenterol. 2024 Nov 1;35(11):811–21. doi: 10.5152/tjg.2024.24110 (PMC11562744; doi:10.5152/tjg.2024.24110)

Supplementary Figure 1. Basic Characteristics of Study.

Author	Year	Country	Duration	Study design		MINORS
Gerges et al.	2022	Germany	Feb.2019 — Jun.2021	Prospective	Multiple-center	15
van der Wiel et al.	2022	Netherlands	Dec.2017 — Jul.2020	Prospective	Single-center	13
Bick et al.	2021	America	Sept.2013 — Sept.2019	Retrospective	Single-center	12
Gutierrez et al.	2019	America	Feb.2015 — Sept. 2017	Retrospective	Multiple-center	8
Gerges et al.	2019	Germany	2015 — 2017	Retrospective	Multiple-center	9
Han et al.	2019	America	Jan.2000 — Jun.2017	Retrospective	Single-center	10
Ogura et al.	2018	Japan	Oct.2016 — Aug.2017	Retrospective	Single-center	8
Canena et al.	2018	Portugal	Jan.2017 — Dec.2017	Prospective	Multiple-center	12
Bekkali et al.	2017	England	Feb.2013 — Apr.2016	Retrospective	Single-center	10
Navaneethan et al.	2016	America	Feb.2015 — Oct.2015	Retrospective	Multiple-center	8
Attwell et al.	2015	America	Oct.2008 — Sept.2011	Retrospective	Multiple-center	11
Attwell et al.	2014	America	Jan.2000 — Mar.2011	Retrospective	Single-center	8
Ito et al.	2014	Japan	May.2005 — Dec.2012	Prospective	Single-center	8
Alatawi et al.	2013	France	2009 — 2010	Retrospective	Single-center	11
Maydeo et al.	2011	India	Mar.2010 — Feb.2011	Prospective	Single-center	12
Fishman et al.	2009	America	NA	Retrospective	Multiple-center	8
Howell et al.	1999	America	NA	Retrospective	Single-center	8

MINORS, Methodological Index for Non-Randomized Studies; NA, no information available

Supplementary Figure 2. Basic information about the operation.

Author	Year	Lithotripsy	No.Intervention	Time (min)
Gerges et al.	2022	EHL & LL	1.4±0.6	31.1±19
van der Wiel et al.	2022	EHL	[#]1	63.8±4.8
Bick et al.	2021	EHL	1.6±0.6	101.6±68.2
Gutierrez et al.	2019	EHL & LL	NA	62.8±23
		EHL	NA	74.4±25.5
		LL	NA	53.8±16.2
Gerges et al.	2019	EHL，LL	NA	NA
Han et al.	2019	EHL，LL	Total：160	NA
		EHL	1.39±0.76	NA
		LL	1.92±1.12	NA
Ogura et al.	2018	EHL	1.31	NA
Canena et al.	2018	EHL，HL	NA	[#]50（45—60）
Bekkali et al.	2017	EHL	NA	48±15
Navaneethan et al.	2016	LL	NA	NA
Attwell et al.	2015	LL	NA	NA
Attwell et al.	2014	EHL，LL，ML	Total：88	NA
Ito et al.	2014	EHL	NA	NA
Alatawi et al.	2013	LL	NA	92.0±27.7
Maydeo et al.	2011	LL	NA	45.9（30—90）
Fishman et al.	2009	EHL	NA	NA
Howell et al.	1999	EHL	1.5±0.55	NA

EHL, Electrohydraulic Lithotripsy ; LL, Laser Lithotripsy ; HL , Holmium Laser ; ML, Mechanical Lithotripsy

Supplementary Figure 3. Forest plot of clinical success with using EHL and with using LL.

- **EHL**

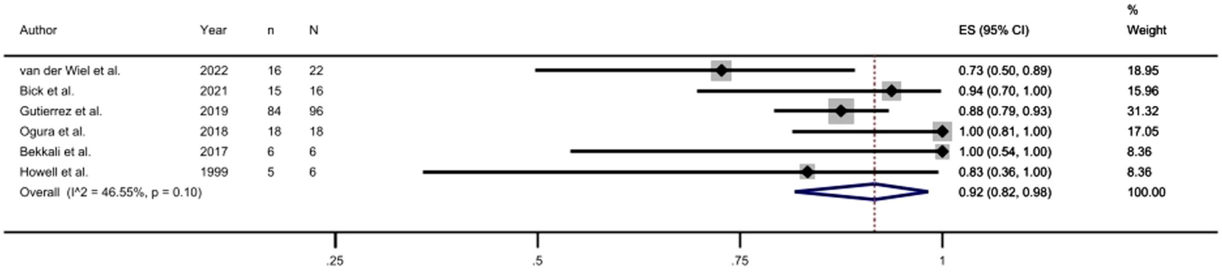

- **LL**

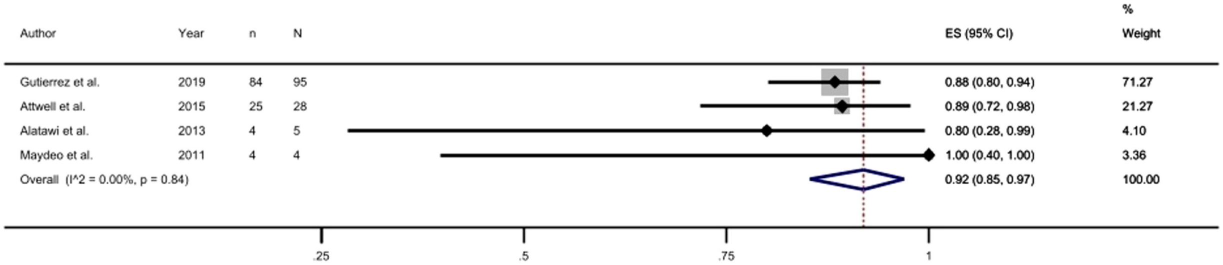

Supplementary Figure 4. Forest plot of adverse events with using EHL and with using LL.

- **EHL**

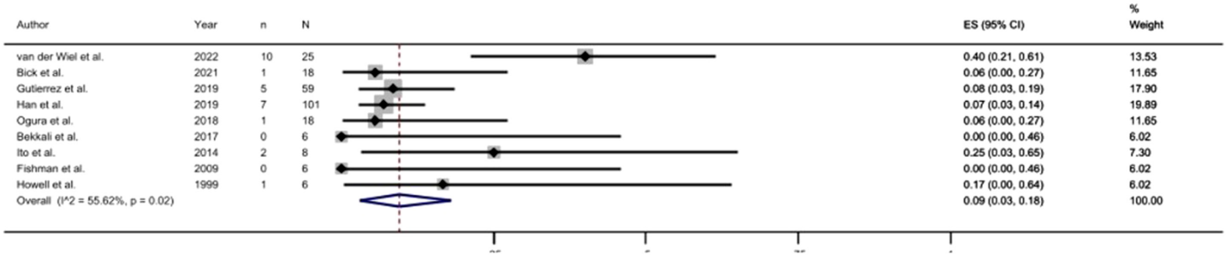

- **LL**

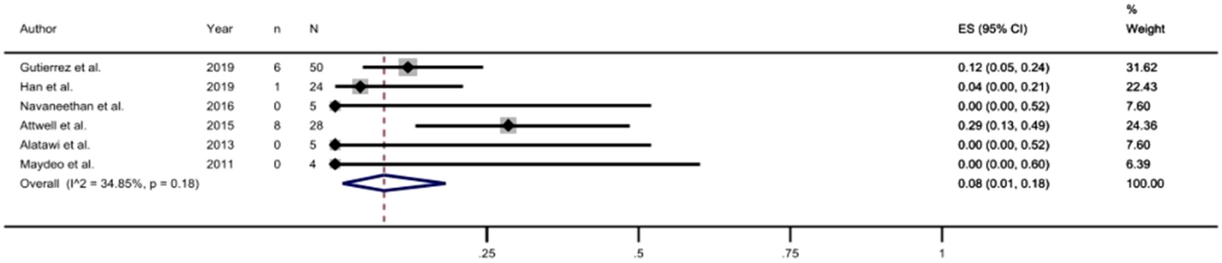

Supplement: Supplementary Material [file supplementary_material.pdf]
